# Berberine enhances L1 expression and axonal remyelination in rats after brachial plexus root avulsion

**DOI:** 10.1002/brb3.1792

**Published:** 2020-08-07

**Authors:** Shuangxi Chen, Bing He, Guijuan Zhou, Yan Xu, Lin Wu, Yangzhi Xie, Yihui Li, Shuangqin Chen, Jianghua Huang, Heng Wu, Zijian Xiao

**Affiliations:** ^1^ The First Affiliated Hospital University of South China Hengyang China; ^2^ Leiyang People's Hospital Leiyang China

**Keywords:** brachial plexus root avulsion, L1CAM, motoneuron

## Abstract

**Background and Purpose:**

Enhanced remyelination of the regenerated axons results in functional re‐innervation and improved functional motor recovery after brachial plexus root avulsion (BPRA). The neural cell adhesion molecule L1 (L1CAM, L1) regulates myelination and promotes regeneration after acute injury in the nervous system. Berberine (BBR) can exert neuroprotective roles against the lesion. Herein, we investigated whether berberine (BBR) can affect the expression of L1 and enhance the axonal remyelination in rats following BPRA.

**Methods:**

The surgical procedures were performed to build the rat brachial plexus avulsion and re‐implantation model, and then, the rats were treated with BBR. After the rehabilitation for 12 weeks, the musculocutaneous nerves were collected for quantitative real‐time PCR, Western blot analysis, and histochemical and immunofluorescence staining.

**Results:**

We observed that, BBR treatment ameliorated the abnormal musculocutaneous nerve fibers morphology, up‐regulated the L1 expression, increased the myelination‐related genes, decreased the differentiated‐associated genes, and up‐regulated the phosphorylation of ERK.

**Conclusion:**

These results suggest that BBR may enhance L1 expression and promote axonal remyelination after spinal root avulsion.

## Graphical abstract

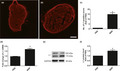

## 1 INTRODUCTION

Brachial plexus root avulsion (BPRA), one of brachial plexus injuries, is considered to be a polytrauma associated with motorbikes (Faglioni, Siqueira, Martins, Heise, & Foroni, 2014), especially happen in young men of reproductive age (Giugale, Henrikson, Baronne, & Lee, 2015). In this traumatic condition, extensive motoneuron death in spinal cord, motor axon degeneration in musculocutaneous nerve, and de‐innervation of targeted muscles were observed, leading to serious functional deficits in the upper limb (Hallin, Carlstedt, Nilsson‐Remahl, & Risling, 1999). Although the injured axons can re‐innervate the target muscle after surgical re‐implantation of the avulsed ventral roots, the functional recovery is still disappointing (Fang et al., 2016). Several combinatorial strategies have been performed for nerve re‐implantation in animal models (Barbizan et al., 2013; Chu, Du, & Wu, 2008). Although some of neurotrophic factors were discovered to stimulate the neural survival and axonal growth, their clinical applications are largely limited due to the tangible ability of through the blood–brain barrier (BBB) caused by their high molecular weight and hydrophility (Konishi, Chui, Hirose, Kunishita, & Tabira, 1993; Oliveira, Risling, Negro, Langone, & Cullheim, 2002; Pardridge, 1998). It is an urgent task to find a medicine, which is effective, safe, and convenient for clinical administration. Also, exploring the injury and theoretic mechanism for the BPRA is necessary.

Attempts to target individual molecules that may promote the motor functional recovery of BPRA have been made to counteract the motor neuron death by enhancing axonal remyelination (Gordon, Sulaiman, & Boyd, 2003; Hoke, 2006). One of these molecules is berberine (BBR), a major bioactive compound naturally extracted from multiple traditional Chinese medicine with long‐lasting effects and low toxicity, including Phellodendri Chinense Cortex, Berberidis Radix, and Coptis Rhizoma (Kumar et al., 2015; Wong, Chin, & Ima‐Nirwana, 2019). BBR promotes the functional recovery after traumatic brain injury and spinal cord injury via regulating oxidative stress and glia‐mediated inflammation (Huang et al., 2018; Wang et al., 2017). BBR promotes neurite extension and axonal regeneration in sciatic nerve injury (Han, Heo, & Kwon, 2012), and ameliorates neuropathic pain in peripheral nerve injury (Yang et al., 2018). Berberine promotes nerve regeneration through IGFR‐mediated JNK‑AKT signal pathway (Zhang et al., 2018). Most importantly, BBR is able to penetrate the blood–brain barrier (BBB) and keep relatively stable in the brain (Tan et al., 2013).

Also, multiple molecular mechanisms need to be taken into consideration to account for the effect of BBR on axonal remyelination in BPRA. We therefore searched for other target molecule that could ameliorate BPRA, preferentially relating to axonal remyelination. One promising recognition molecule in the diseased nervous system is L1 cell adhesion molecule (L1CAM), a membrane glycoprotein first described in 1984 (Rathjen & Schachner, 1984). In vitro and in vivo studies revealed that L1 modulates neurite outgrowth, neuronal survival, and neuritogenesis (Chen et al., 2020; Chen, Hu, Liao, & Zhao, 2015; Lemmon, Farr, & Lagenaur, 1989; Wei & Ryu, 2012). Previous studies have also found that L1 regulates the expression levels of ChAT and influences ChAT activity (Cui et al., 2011). L1 expression exerts essential roles in memory, learning, and regeneration following lesion (Chaisuksunt et al., 2000; Gu et al., 2005; Liljelund, Ghosh, & van den Pol, 1994; Luthl, Laurent, Figurov, Muller, & Schachner, 1994). Myelin basic protein cleaves cell adhesion molecule L1 and improves regeneration after injury (Lutz et al., 2016). Adhesion molecule L1 overexpressed under the control of the neuronal Thy‐1 promoter improves myelination after peripheral nerve injury in adult mice (Guseva et al., 2011). Schwann cells engineered to express the cell adhesion molecule L1 accelerate myelination and motor recovery after spinal cord injury (Lavdas et al., 2010).

Previous study has reported that BBR enhances survival and axonal regeneration of motoneurons following spinal root avulsion and re‐implantation in rats (Zhang et al., 2019); however, whether BBR can regulate axonal remyelination in musculocutaneous nerve to promote motor function recovery and the underlying mechanism remain unclear. Given the key roles of L1 in regeneration and myelination, we were interested in which extent BBR modulates L1 expression in BPRA. Regarding this spinal cord injury, we hypothesized that BBR may affect L1 expression and exert a protective role in MNs by fostering axonal remyelination ‐related signaling in mice following BPRA. Here, we reported on the neuroprotective role of BBR on the treatment of spinal root avulsion and the modulating role on L1 expression.

## 2 MATERIALS AND METHODS

### 2.1 Animals

200–220 g male rats purchased from the Hunan Medical Laboratory Animal Center (PR China) were maintained at 22°C on a 12‐hr light/12‐hr dark cycle and provided food and water ad libitum. The Laboratory Animal Ethics Committee at University of South China approved all experimental protocols.

### 2.2 Brachial plexus avulsion and re‐implantation model

The surgical procedures performed on animals were similar to previously described with minor modifications (Chen et al., 2019; Li et al., 2015). The animals anesthetized by intraperitoneal injection of chloral hydrate at a concentration of 300 mg/kg were placed on the surgical table with the prone position. After right C4 to C6 hemilaminectomies, the C5‐7 segments of spinal cord were identified. After opening the dura mater, the right C5‐7 dorsal and ventral roots were dissected out and avulsed to avoid re‐innervation. For the re‐implantation models, the C6 ventral root was replanted to the exact point of detachment (on the surface of piamater) immediately following the avulsion (Chai, Wu, So, & Yip, 2000). Muscles and skin were sutured in layers after the surgical procedures. The animals were returned to their cages after recovering from anesthesia.

### 2.3 Treatment and grouping

The rats (*n* = 15/group) with re‐implantation were randomly divided into two groups: (a) PBS and (b) BBR (Shifeng Biotechnology Ltd.). The treatment groups were treated daily with BBR (500 μl at concentration of 4 mg/ml) or vehicle (5% DMSO in PBS, 500 μl), by subcutaneous injection near the injury site daily for 7 days after surgery. The duration for rehabilitation was 12 weeks, and none of the animals died during that time.

### 2.4 Tissue preparation

Rats were killed by decapitation after isoflurane anesthesia. The musculocutaneous nerves were collected.

For qPCR analysis, total RNA was extracted from nerve sections using TRIzol reagent according to the manufacturer's protocol (Invitrogen). And the following steps were performed following the previous paper (Fang et al., 2016).

For Western blot analysis, musculocutaneous nerves were dissolved in 100 μl RIPA buffer with 1% PMSF (P8340‐1, Solarbio Bioscience & Technology). After being homogenized by a microtissue grinder (749540‐0000, Kimble Chase Life Science and Research Products, LLC), the supernatants were collected after centrifugation at 14,000 *g* for 15 min at 4°C.

For histological staining, mice were deeply anesthetized with isoflurane at the end of survival period (12 weeks post surgery) and transcardially perfused with saline followed by 4% paraformaldehyde (PFA) in 0.1 M PBS (pH 7.4) through the left cardiac ventricle. The musculocutaneous nerve was dissected and harvested for further analyses. After being postfixed in 4% PFA for 24 hr at 4°C, tissues were transferred into a solution of PBS containing 15% and 30% sucrose for 24 hr at 4°C, respectively. After being sunk, the tissues were cut into sections on a sliding microtome (LEICA CM1950, Leica).

### 2.5 Quantitative real‐time PCR

Quantitative real‐time PCR was performed using SYBR Green Kit (Takara) in an iCycler iQTM (Bio‐Rad) according to the standard protocols and the previous paper (Fang et al., 2016). The primer sequences were for quantitative real‐time PCR were listed in Table 1.

**TABLE 1:** The primer sequences were for quantitative real‐time PCR

Name	Toward	Sequences (5′−3′)
L1	Forward	GGGACCTACAGCCTGACACCAAA
	Reverse	AGCACTGACAAAGGCGATGAACCA
hmgcr	Forward	ACATACTGGACTGAAACACGGGCAT
	Reverse	AGAACACGGCACGGAAAGAACCAT
prx	Forward	ACCTTCCACATCTCATTGCCT
	Reverse	CTTGAGTTTGTGCCCGCCAT
mpz	Forward	TCCTCGGGCTCAAATCCACA
	Reverse	ACGTCATTGGTCCTCGGTCA
egr2	Forward	CCCCTCCGCTCACGCCACT
	Reverse	ACCCTCACCGCCTCCACTTGC
pmp22	Forward	CCTACAGCAGAACAGAGACCCGAT
	Reverse	TCCTGATGCTCCGACCGTGA
Pou3f1	Forward	CCTGTTTCCCTACCGCTTCC
	Reverse	GGAGAACAACCCAGAAAGCCAAA
ngfr	Forward	ACGACCAGCAGACCCATACGC
	Reverse	ATGTCGCCAGGTATCCCCGTT
β‐actin	Forward	ACATCCGTAAAGACCTCTATGCC
	Reverse	TACTCCTGCTTGCTGATCCAC

### 2.6 Western blot analysis

Western blot analysis was performed as previous studies with minor modifications (Li et al., 2017; Xu et al., 2017).

The tissue lysates from musculocutaneous nerves mixed with a sample loading buffer (0.125 M Tris–HCl, pH 6.8, 20% glycerol, 10% sodium dodecyl sulfate, 0.1% bromophenol blue, and 5% β‐mercaptoethanol) were heated at 95°C for 15 min. Protein samples were subjected to 10% SDS–PAGE and electroblotted onto polyvinylidene difluoride (PVDF) membranes (Millipore). After being incubated in 5% bovine serum albumin (BSA) diluted in Tris–HCl saline buffer supplemented with 0.1% Tween‐20 (TBST, pH 7.4) for 1 hr to block nonspecific protein binding sites, membranes were incubated overnight at 4°C with following primary antibodies: goat anti‐L1 antibody (1:1,000; AF277, R&D system), rabbit anti‐pERK1/2 (1:1,000; ab4370, Abcam), or rabbit anti‐ERK1/2 (1:1,000, ab4695, Abcam), and mouse anti‐GAPDH (1:1,000; SC‐365062, Santa Cruz Biotechnology). Wash the membrane with 0.1% TBST 3 times for 5 min each at RT, horseradish peroxidase‐conjugated goat anti‐rabbit secondary antibodies (1:2,000; ab6721, Abcam) goat anti‐mouse secondary antibodies (1:2,000; ab97023, Abcam), or donkey anti‐goat (1:2,000; ab6885, Abcam) secondary antibodies, diluted in TBST were incubated at RT for 1.5 hr. Next, membranes were washed in 0.1% TBST 3 times for 5 min each at RT. The immunoreactive bands were visualized by an enhanced chemiluminescence (ECL) kit (170‐5061, Bio‐Rad Laboratories). The signal intensities were quantified by ImageJ 5.0 software.

### 2.7 Histochemical and immunofluorescence staining

Histochemical and immunofluorescence staining were performed as previous studies with minor modifications (Chen et al., 2019; Jiang et al., 2016).

For Luxol fast blue (LFB) staining, nerve sections at the thickness of 3 µm were immersed in LFB solution at 60°C for 12 hr and then sequentially washed with 95% ethanol, water, 0.1% lithium carbonate solution, 70% ethanol, and water. Next, the tissue slides were dehydrated with 95% ethanol and 100% ethanol, rinsed with xylene, and mounted with coverslide using neutral balsam.

Hematoxylin and eosin (H&E) staining was performed to assess the fibrosis. Cross sections of musculocutaneous nerve were subjected to H&E staining. Briefly, nuclei were stained with Harris hematoxylin for 8 min, followed by differentiation with 0.3% acid alcohol for several seconds. Next, cytoplasm was stained with eosin for 2 min. Sections were dehydrated by graded ethanol (95%, 95% and 100%, 2 dips each; 100% for 2 min; 100% for 12 min). Images used for observation were digitalized by light microscopy (MBF Nikon Microscope).

For immunofluorescence staining, the tissue slides were blocked with 10% normal donkey serum (NDS) in PBS at RT for 1 hr. Samples were incubated at 4°C overnight with a mixture of following primary antibodies: goat anti‐L1 antibody (1:200; AF277, R&D system), rabbit anti‐MBP (1:200, M3821, Sigma), and mouse anti‐S100 (1:200, ab4066, Abcam). After rinsing with PBS, immunoreactivities were visualized by incubation with Alexa fluor 488 or 546 fluorescent secondary antibodies (1:1,000, Invitrogen). The samples mounted using ProLong^®^ Gold Anti‐fade reagent with 4′,6‐diamidino‐2‐phenylindole (DAPI; P36935, Gibco; Thermo Fisher Scientific, Inc.) were imaged using an AxioObserver A1 microscope (Carl Zeiss) with and AxioVision 4.6 software (Carl Zeiss).

### 2.8 Cell counts

The number of LFB, L1, MBP, and S100‐positive axons, fibroblast nuclei was calculated using ImageJ 5.0 software. This step was carried out by two people who were blinded to the treatment group of animals.

### 2.9 Statistical analysis

All statistical analyses were performed using GraphPad Prism6 software. Data were expressed as mean ± *SEM* and performed using Student's *t* test. *p* < .05 was considered statistically significant.

## 3 RESULTS

### 3.1 BBR ameliorates the morphology of the abnormal musculocutaneous nerve fibers after BPRA

To assess the therapeutic effect of BBR on the morphologic improvement of the nerve in injured mice, the changes in the nerve fibers were analyzed by LFB and H&E staining.

**FIGURE 1:**
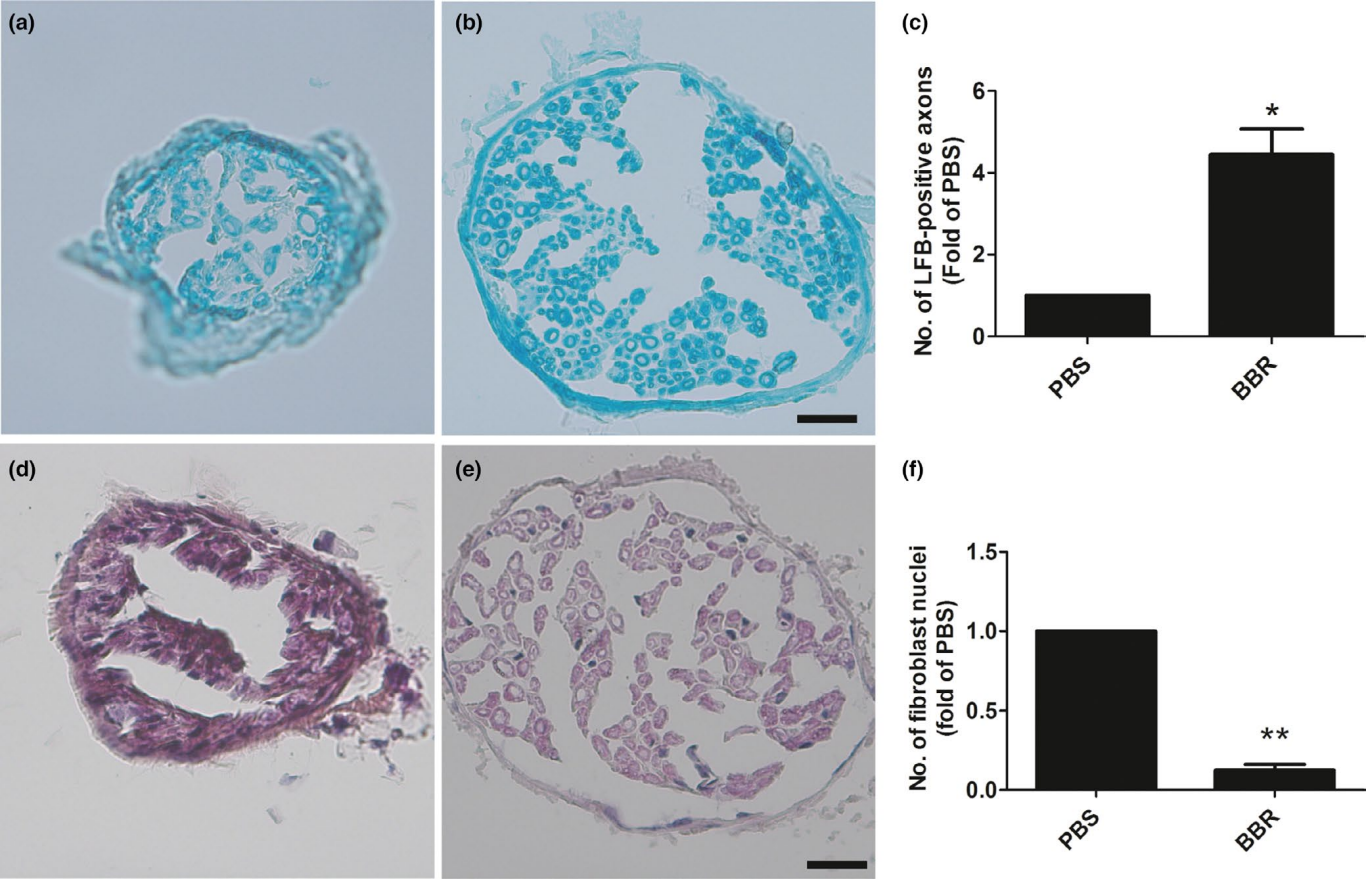
BBR ameliorates the morphology of the abnormal musculocutaneous nerve fibers after BPRA in rats. (a–c) The number of LFB‐positive axons is increased, and (d–f) the number of fibroblast nuclei is decreased after BBR treatment. Scale bars represent 20 μm. *p < .05,**p < .01, *n* = 3/subgroup

We found that, in the PBS‐treated group, scarce nerve fibers or degenerative, disorganized were observed; however, these abnormalities were dramatically ameliorated after treatment with BBR. Also, the number of LFB‐labeled axons in the PBS‐treated group was much lower than that in the BBR‐treated group (Figure 1a–c). According to the statistical analysis of fibroblast nuclei evaluation, the amounts of fibroblasts in the BBR‐treated group were significantly decreased compared with those in the PBS‐treated group (Figure 1d–f).

These results demonstrated that BBR ameliorates the morphology changes of the musculocutaneous nerve in mice with BPRA and maintains its structural integrality.

### 3.2 BBR enhanced L1 expression in musculocutaneous nerve after BPRA

To investigate the effect of BBR on the L1 expression in musculocutaneous nerve, immunohistochemical staining, qPCR, and Western blot were performed.

**FIGURE 2:**
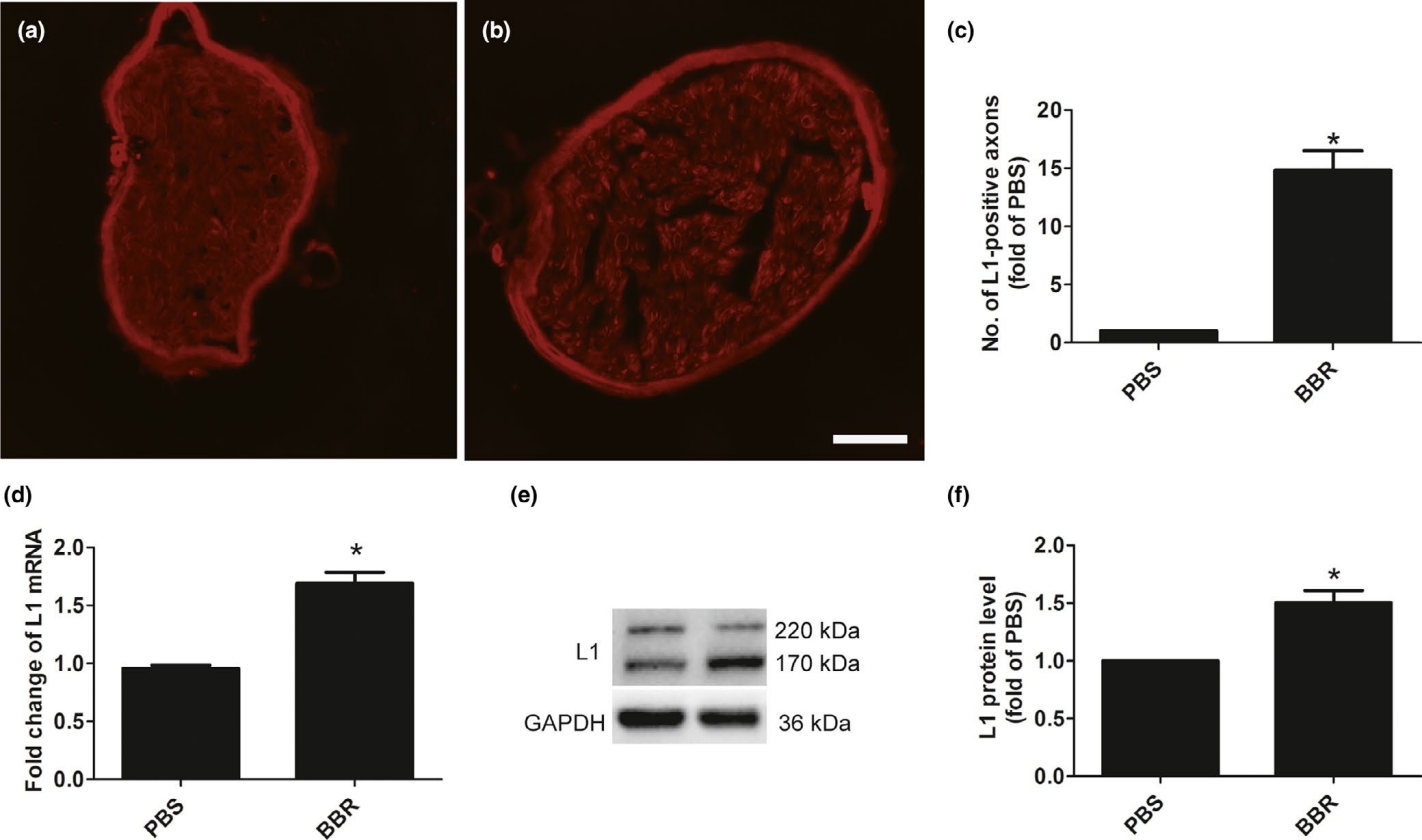
Effect of BBR on the L1 changes in musculocutaneous nerve fibers after BPRA after BPRA in rats. BBR increased the L1‐positive axons (a–c), L1 (d) mRNA, and protein levels (e,f). Scale bar represents 20 μm. *p < .05, *n* = 3/subgroup

We found that, the number of L1‐positive axons in the PBS‐treated group was lower than in the BBR‐treated group (Figure 2a–c). Also, treatment of BBR can up‐regulate the L1mRNA and protein levels (Figure 2d–f).

These results suggested that BBR may exert functional roles in BPRA via regulating L1.

### 3.3 BBR promoted the remyelination in musculocutaneous nerve after BPRA

To investigate the effect of BBR on the remyelination in musculocutaneous nerve after injury, immunohistochemical staining, qPCR was performed to test the change of genes.

**FIGURE 3:**
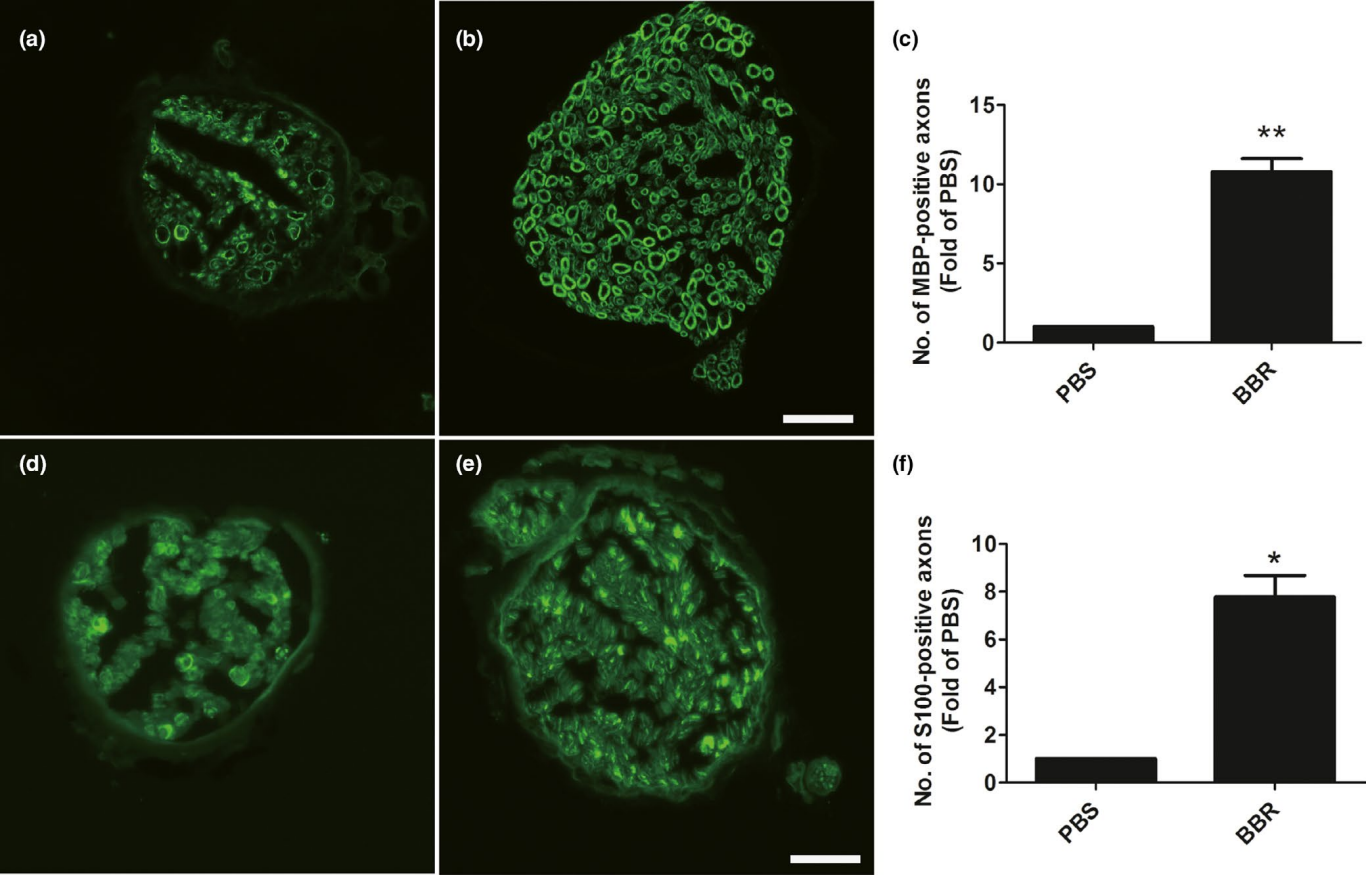
BBR up‐regulates the MBP and S100 expression levels in musculocutaneous nerve after BPRA in rats. The number of MBP‐positive axons (a–c) and S100‐positive axons (d–f) is increased in response to BBR treatment. Scale bars represent 20 μm. *p < .05,**p < .01, *n* = 3/subgroup

We found that, the number of L1‐positive axons in the PBS‐treated group was lower than in the BBR‐treated group (Figure 3a–c). Also, treatment of BBR can up‐regulate the L1mRNA and protein levels (Figure 3d–f).

**FIGURE 4:**
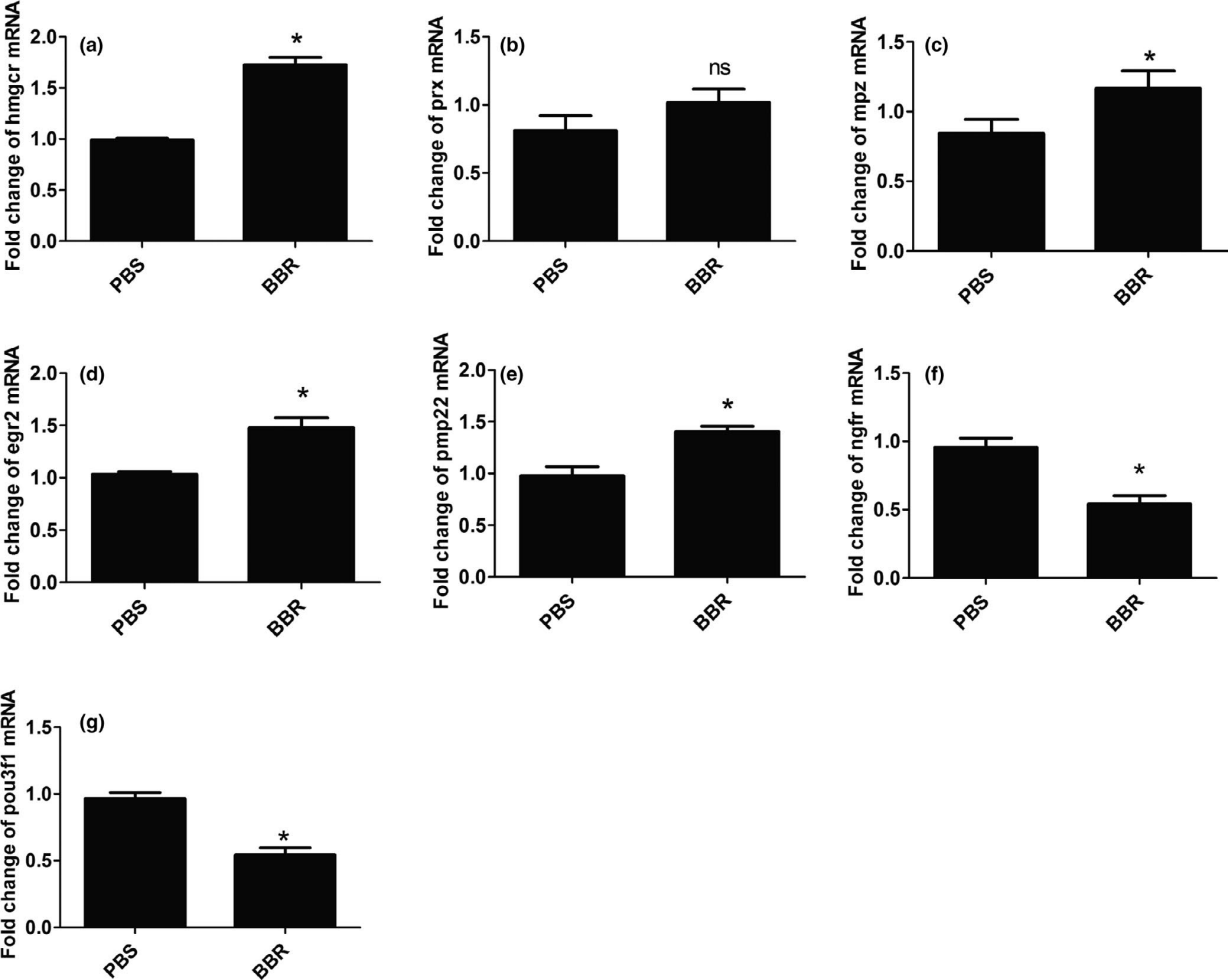
BBR up‐regulates the mRNA levels of myelination‐associated genes in musculocutaneous nerve after BPRA in rats. The mRNA levels of myelination‐associated genes (hmgcr, mpz, prx, egr2, pmp22) are increased (a–e), and dedifferentiation‐associated genes (ngfr, pou3f1) were down‐regulated (f,g) in response to the treatment of BBR. *p < .05, *n* = 3/subgroup

We also observed that, the myelination‐related genes (hmgcr, mpz, prx, egr2, pmp22) were up‐regulated (Figure 4a–e), and dedifferentiation‐associated genes (ngfr, pou3f1) were down‐regulated (Figure 4f,g) in response to the treatment of BBR.

These results suggested that BBR may exert functional roles in BPRA via promoting remyelination.

### 3.4 BBR promoted the ERK phosphorylation in musculocutaneous nerve after BPRA

To investigate the effect of BBR on the ERK phosphorylation in musculocutaneous nerve after injury, Western blot was performed.

**FIGURE 5:**
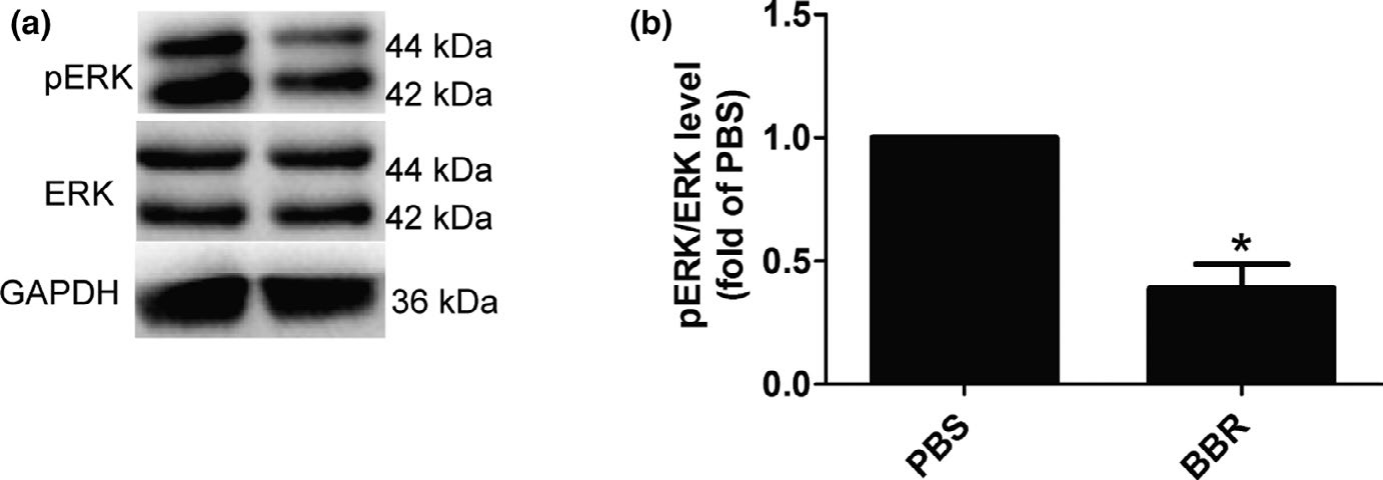
BBR down‐regulates the phosphorylation levels of ERK in musculocutaneous nerve after BPRA in rats. ERK phosphorylation levels (a,b) were down‐regulated in response to the treatment of BBR. *p < .05, *n* = 3/subgroup

We observed that, the ERK phosphorylation was down‐regulated in response to the treatment of BBR (Figure 5a,b).

These results suggested that BBR may exert functional roles in BPRA via inhibiting ERK activity.

## 4 DISCUSSION

When avulsion occurs, motor neurons degenerate progressively in the avulsed spinal segment (Koliatsos, Price, Pardo, & Price, 1994; Wu, 1996) and most of affected motor neurons finally die (Gu et al., 2004), causing the paralysis of the target muscle groups. To accelerate motor recovery after BPRA, multiple surgical approaches have been carried out in animal models. Re‐implantation surgery which is a widely performed technique is not able to restore motor function completely mainly because the axonal growth of spinal MNs is too slow to re‐innervate the target muscles before the atrophy happen (Carlstedt, 2008; Carlstedt, Anand, Htut, Misra, & Svensson, 2004). The biceps are innervated only by the musculocutaneous nerve. In order to restore motor function, injured MNs have to survive and regenerate axons (Carlstedt et al., 2004; Wu & Li, 1993). Due to the essential role of MNs survival in functional recovery, early and effective strategies to promote the survival of lesioned MNs are necessary for prolonging the time window for BPRA treatment (McKay Hart, Brannstrom, Wiberg, & Terenghi, 2002). Multiple combinatorial means have been attempted in animal models with root avulsion injuries following re‐implantation (Barbizan et al., 2013; Chu et al., 2008). In the previous study, BBR promotes the motor function recovery via enhancing survival and axonal regeneration of motoneurons (Zhang et al., 2019); in the current study, we revealed that BBR enhanced L1 expression and remyelination in musculocutaneous nerve in rats after BPRA.

L1 exerted essential roles in the survival of cultured dopaminergic neurons and in the substantia nigra of a mouse model of Parkinson's disease was considered to be neuroprotective for all of neurons investigated so far (Dihne, Bernreuther, Sibbe, Paulus, & Schachner, 2003). Overexpressed L1 in transfected embryonic stem cells supported re‐growth and decreased the dying‐back of axons in corticospinal tract of adult mice with spinal cord lesion (Chen, Bernreuther, Dihne, & Schachner, 2005; He et al., 2012). In acute and chronic injuries of the central nervous system of adult mammals, which are reduced from regeneration in the inhibitory tissue environment, L1 promotes regeneration‐conducive processes (Chen et al., 2007; Roonprapunt et al., 2003). After peripheral nerve injury and spinal cord injury, L1 improves myelination and accelerates motor recovery (Guseva et al., 2011; Lavdas et al., 2010). In the present study, we observed that BBR up‐regulated L1 expression after BPRA.

The formation of myelin depends on the interaction between the axons and Schwann cells. After peripheral nerve injury, Schwann cells are released from the degenerating nerve, dedifferentiated, and then actively participated in axonal regeneration(Namgung, 2014). Therefore, BBR may affect one or both of these aspects to increase remyelination. In the present study, we observed that BBR up‐regulated MBP and S100 expression levels, and the myelination‐related genes, and down‐regulated the dedifferentiation‐associated genes.

It has been previously reported that Mek‐Erk signaling was implicated in mediating Schwann cell dedifferentiation and myelin breakdown after acute nerve injury (Napoli et al., 2012). Also, sustained MAPK/ERK inactivation in adult schwann cells promotes nerve repair (Cervellini et al., 2018). In the present study, we observed that BBR inhibited the ERK phosphorylation.

## 5 CONCLUSION

Taken together, we preliminarily hypothesize that treatment of BBR may promote remyelination of regenerating axons after BPRA via regulating L1. These observations potentially support a novel insight into the treatment of multiple disorders tightly associated with L1 malfunction.

Although the results in the present study look promising, our study exhibited several limitations: More sophisticated approach, such as using the L1 siRNA, should be performed to gain insight into the relationship between BBR and L1 in BPRA pathology.

## CONFLICT OF INTEREST

None declared.

## AUTHOR CONTRIBUTION

ZX, HW, and SC conceived and designed the experiments. SC, BH, GZ, YX, LW, YX, YL, SC, and JH performed the experiments and analyzed the data. SC and BH contributed reagents/materials/analysis tools. ZX, HW, and SC wrote the paper.

### Peer Review

The peer review history for this article is available at https://publons.com/publon/10.1002/brb3.1792.

## Data Availability

The data that support the findings of this study are available from the corresponding author upon reasonable request.
